# Patients with Poor Response to Antipsychotics Have a More Severe Pattern of Frontal Atrophy: A Voxel-Based Morphometry Study of Treatment Resistance in Schizophrenia

**DOI:** 10.1155/2014/325052

**Published:** 2014-07-23

**Authors:** Mario Quarantelli, Olga Palladino, Anna Prinster, Vittorio Schiavone, Barbara Carotenuto, Arturo Brunetti, Angela Marsili, Margherita Casiello, Giovanni Muscettola, Marco Salvatore, Andrea de Bartolomeis

**Affiliations:** ^1^Biostructure and Bioimaging Institute, National Research Council, Via T. De Amicis 95, 80145 Naples, Italy; ^2^Section of Psychiatry, Department of Neuroscience and Reproductive and Odontostomatological Sciences, University “Federico II”, 80131 Naples, Italy; ^3^IDC Hermitage Capodimonte Spa, 80145 Naples, Italy; ^4^Department of Advanced Biomedical Sciences, University “Federico II”, 80131 Naples, Italy; ^5^Department of Neuroscience, Reproductive and Odontostomatological Sciences, University “Federico II”, 80131 Naples, Italy

## Abstract

Approximately 30% of schizophrenia patients do not respond adequately to the therapy. Previous MRI studies have suggested that drug treatment resistance is associated with brain morphological abnormalities, although region-of-interest analysis of MR studies from nonresponder and responder patients failed to demonstrate a statistically significant difference between these two schizophrenia subgroups. We have used a voxel-based analysis of segmented MR studies to assess structural cerebral differences in 20 nonresponder and 15 responder patients and 16 age-matched normal volunteers. Differences between the three groups emerged bilaterally mainly at the level of the superior and middle frontal gyri, primarily due to reduced grey matter volumes in nonresponders, as compared to both normal volunteers and responder patients. Post hoc direct comparison between the two schizophrenia subgroups demonstrated significantly reduced grey matter volumes in middle frontal gyrus bilaterally, in the dorsolateral aspects of left superior frontal gyrus extending into postcentral gyrus and in the right medial temporal cortex. Our results extend and integrate previous findings suggesting a more severe atrophy in nonresponder schizophrenia patients, compared to responder patients, mainly at the level of the superior and middle frontal gyri. Longitudinal studies in drug-naïve patients are needed to assess the role of these associations.

## 1. Introduction

Antipsychotics represent the mainstay of schizophrenia pharmacological treatment; however, approximately 20% to 35% of people affected by schizophrenia, under optimal antipsychotic treatment and when all major cofactors are controlled for, fail to respond to antipsychotics [1–6]. Treatment-resistant schizophrenia patients show a reduced quality of life, increased drug abuse [7], and reduced cognitive performance compared to patients responders to the treatment [8].

Understanding the mechanisms of treatment response is relevant to schizophrenia pathophysiology and to the therapeutic implications. However, treatment-resistant schizophrenia, since its initial definition introduced by Kane and collaborators in the Multicenter Clozapine Trial [9], remains a post hoc diagnosis based on the clinical course. Clinical features at diagnosis such as poorer premorbid function, an earlier age at onset of positive symptoms, male gender, family history of schizophrenia, longer duration of untreated psychosis, severe negative symptoms, presence of soft neurological signs, absence of precipitating factors, and a history of substance abuse can only partially predict resistance to the treatment [10–13].

Although it has been speculated that brain imaging, both structural and functional, could contribute to the identification of biological variables related to treatment response or resistance, to the best of our knowledge there are only a few imaging studies on the putative structural correlates of drug-resistance [14–22], which overall suggest that a more severe pattern of brain alterations may underlie treatment resistance.

In this study, we tested the hypothesis that brain regional abnormalities may correlate with treatment resistance in schizophrenia patients. We applied a voxel-based analysis of segmented MRI images (brain-wise analysis, not based on predefined ROIs) to assess brain structural differences between NonResp-SC and Resp-SC patients and compare both groups with normal control.

## 2. Material and Methods

### 2.1. Subjects

Fifty-one subjects were studied. Fifteen Resp-SC and twenty NonResp-SC male patients were sequentially enrolled among the patient population referring to the Psychiatry Section, Unit of Treatment Resistant Psychosis at the Department of Neuroscience of the University of Naples Federico II.

The patients were referred to the unit by psychiatrists of Community Health Centers, private practice psychiatrists or by general physicians. All the NonResp-SC patients had been already treated with at least two antipsychotic trials when first admitted at Outpatient Clinics for treatment-resistant psychosis.

Exclusion criteria for patients were left-handedness; age below 18 years; evidence of cardiovascular, metabolic, or neurological impairment; previous head injuries requiring hospitalization; history of mental retardation, alcoholism, substance dependence over the previous 3 years or abuse over the previous 6 months; head injury or electroconvulsive therapy; and lack of willingness to participate to the study.

Sixteen age-matched male normal volunteers (NV) were also enrolled over the same timeframe through local advertising. Exclusion criteria for NV were evidence of cardiovascular, metabolic, neurological, and psychiatric impairment; previous head injuries requiring hospitalization; alcohol or recreational drugs use; or treatment with medications active on the CNS.

All patients underwent clinical assessment to confirm the diagnosis of schizophrenia according to DSM IV TR. The assessment was carried out by a psychiatrist (MC) with more than 20 years of experience in psychosis diagnosis and treatment and was confirmed by a second psychiatrist (GM) by means of a Structured Treatment resistant Record Chart, specifically developed at Unit of Treatment Resistant Psychosis.

The Expanded Brief Psychiatric Rating Scale (E-BPRS [25]) was administered to the patients on the same day of the MRI study.

Clinical data were collected from clinical records of the Outpatient Clinics for Drug Resistance at the Department of Neuroscience, including also patient family interviews, and the duration of psychosis (disease duration—DD) and the duration of untreated psychosis were calculated from the first manifestation of psychotic symptoms such as delusions, hallucinations, thought disorder, or inappropriate/bizarre behavior lasting throughout the day for several days or several times a week and requiring an unambiguous psychiatric intervention [26].

Patients were classified as NonResp-SC based on the lack of a satisfactory clinical improvement despite the sequential use of the recommended doses for 6 to 8 weeks of at least two antipsychotics where at least one of which is atypical [27].

Lack of clinical improvement was defined by all of the following conditions:

1. lack of ≥20% improvement and persistence of a value ≥45 in the total score of the 18-items version of the BPRS (included in the E-BPRS) [9];
2. a score ≥4 in at least two out of the four E-BPRS psychotic items.

In addition to the above criteria, the presence of poor psychosocial and community functioning, which persisted for at least two years despite trials of medication that have been adequate in terms of dose, duration, and adherence was required [28].

The compliance was ascertained for each patient according to the following check points [29]:

1. clinical records of previous psychiatric assessments (either as outpatient or as inpatient)
2. interview with patient and family member or caregivers (that was systematically available for all patients enrolled in the study) addressing systematically the following issues:
  - timing of therapy administration;
  - How many times in a week the medicine was eventually missed (we excluded patients who missed the medicine more than seven times in one month or for three days in row for more than twice in the last month before the assessment);
  - Attitude of the patient toward the medicine: expectation toward disease control, acceptance of potential or actual side effects, and willing to take the therapy after resolution of acute psychotic episode;
  - Co-occurrence of prolonged other medicines administration that could influence, by pharmacokinetic interference, the efficacy of antipsychotics (i.e., polytherapy such as carbamazepine plus haloperidol) and could be responsible for symptoms rebound.

All enrolled patients were under antipsychotic treatment at the time of the MRI scan. Current treatments included haloperidol, olanzapine, aripiprazole, bromperidol, and risperidone in Resp-SC and haloperidol, olanzapine, clozapine, quetiapine, paliperidone, risperidone, clotiapine, and perfenazine in NonResp-SC. The total daily doses of antipsychotics, converted in chlorpromazine mg equivalents per day [23], reported in Table 1, were significantly higher in NonResp-SC patients (*P* = 0.003 at Mann-Whitney test).

Concomitant treatments included selective serotonin reuptake inhibitors (in 1 Resp-SC and 4 NonResp-SC), benzodiazepines (in 3 Resp-SC and 10 NonResp-SC), antiparkinsonian drugs (in 2 Resp-SC and 4 NonResp-SC), and anticonvulsants (in 5 NonResp-SC).

Demographic and clinical data of patient and NV groups are summarized in Table 1. No significant difference was present at ANOVA among the three groups (NV, Resp-SC, and NonResp-SC) in terms of age or between the two SC subgroups in terms of DD and age at onset (AAO).

The two SC subgroups were significantly different in terms of total E-BPRS score (*P* < 10^−9^ at Mann-Whitney test), with significantly higher scores in the NonResp-SC group; this difference was significant also when assessing separately the E-BPRS subscores related to positive psychotic symptoms (BPRS-PS comprising the hallucinatory behavior, unusual thought content, suspiciousness, and conceptual disorganization [30, 31] and mannerism items [32]; *P* < 10^−8^) and negative symptoms (BPRS-NS comprising the blunted affect, emotional withdrawal and motor retardation items [32–34]; *P* < 10^−7^).

The work was carried out in accordance with The Code of Ethics of the World Medical Association (Declaration of Helsinki) for experiments involving humans. All participating subjects gave written consent after the purpose and methods of the study had been explained to them and/or to their legal representatives, and the ethical committees of the participating institutions approved the protocol.

### 2.2. MR Studies and Segmentation

For segmentation purposes, T1-weighted volumes were acquired at 1.5 Tesla (Achieva, Philips Medical Systems, Eindhoven, The Netherland) using a magnetization-prepared 3D fast Gradient-Echo sequence (TR/TE/TI 11/2/600 ms, voxel size 0.98 × 0.98 × 1.2 mm, 124 contiguous axial slices covering the entire brain).

All scans were performed on the same MR scanner, and no HW/SW upgrade was carried out on the scanner during the study.

SPM5 (Wellcome Department of Cognitive Neurology, London, UK, http://www.fil.ion.ucl.ac.uk/spm/software/spm5/) was used to segment the T1-weighted volumes into GM, WM, and CSF probabilistic maps, using the unified segmentation approach [35], which is a fully automated procedure combining within a single framework bias correction, template registration, and tissue classification, thus obviating the need for iterative steps including generation of a site-and study-specific MRI template.

### 2.3. Statistical Analysis: Global Tissues

Following segmentation, for each study, the GM, WM, and CSF volumes were calculated (for each tissue, as the sum of the corresponding probabilities multiplied by voxel volume, only in voxels where the cumulated probabilities of GM, WM and CSF exceeded 50%).

Differences in global brain tissue volumes among the three groups were assessed by general linear model multivariate analysis including total intracranial volume (ICV, the sum of GM, WM, and CSF volumes) and age in the model. Subsequently, only tissues that exhibited a significant difference among groups at MANOVA were tested by three post hoc linear regression analyses of significant main effects (NV versus NonResp-SC, NV versus Resp-SC, and Resp-SC versus NonResp-SC), to localize differences among groups.

### 2.4. Morphometric Analysis

For subsequent voxel-based analysis, GM volumes were normalized to the Montreal Neurological Institute (MNI) space using the SPM mean GM template with 16 nonlinear iterations using 6 × 8 × 6 basis functions to account for global shape differences [36]. Normalized images were resampled by trilinear interpolation to 2 × 2 × 2 mm voxel size. To ensure that the total amount of GM in each region remained unchanged after the warping inherent to spatial normalization, thus allowing subsequent testing for voxelwise differences in the relative volume of GM [37], modulation of normalized GM maps was performed by multiplying the voxel values by the Jacobians derived from the corresponding spatial normalization parameters [38].

Finally, modulated volumes were smoothed with a 5 mm FWHM 3D isotropic Gaussian filter. Local differences in gray matter volume between the three groups were assessed using permutation tests [39, 40] implemented in the Cambridge Brain Analysis software (CamBA version 2.3.0; http://www.bmu.psychiatry.cam.ac.uk/software) running under Linux.

This nonparametric method of analysis allows us to test the null hypothesis of no differences in regional brain tissue volumes between different groups, at the level of spatially contiguous 3D voxel clusters, thus incorporating spatial information, and has been shown to be generally more powerful than other tests, such as those informed only by data at the single voxel level [39–41].

Accordingly, an analysis of covariance (ANCOVA) model was fitted for each intracerebral voxel in the standard space, included in the model as covariates age and ICV. A preliminary voxelwise omnibus ANCOVA, including ICV and age as covariates, was performed to localize relative GM group differences among the three groups. Subsequently, three post hoc analyses of significant main effects (NV versus Resp-SC, NV versus NonResp-SC, and Resp-SC versus NonResp-SC) were performed, restricted to voxels significantly different among the three groups at the omnibus test.

For each post hoc test both direct and inverse contrasts were probed.

For each analysis, a preliminary probability threshold (*P* < 0.05) was applied to the corresponding voxel statistic maps and subthreshold voxels were set to 0, thus creating a set of suprathreshold voxel clusters. The sum of the suprathreshold voxel values (cluster mass, M) was then tested against the M distribution obtained from 10 random permutations of the data sets. Probability thresholds for cluster testing were then set so that the average number of false-positive clusters expected per map was less than one [39, 40].

Clusters showing significant between group differences were localized based on their coordinates in the MNI space [24].

## 3. Results

### 3.1. Global Brain Tissue Volumes

Analysis of global brain tissue volumes showed a significant group effect at multivariate analysis (*P* < 0.05) for global GM volume, corrected for age and intracranial volume (ICV). Post hoc pairwise comparisons showed the effect on GM variance to be due to reduced GM volumes in the NonResp-SC (*P* = 0.03) compared to NV, while no significant differences emerged between Resp-SC and NV or between the two SC subgroups.

### 3.2. Morphometric Analysis

**Figure 1:**
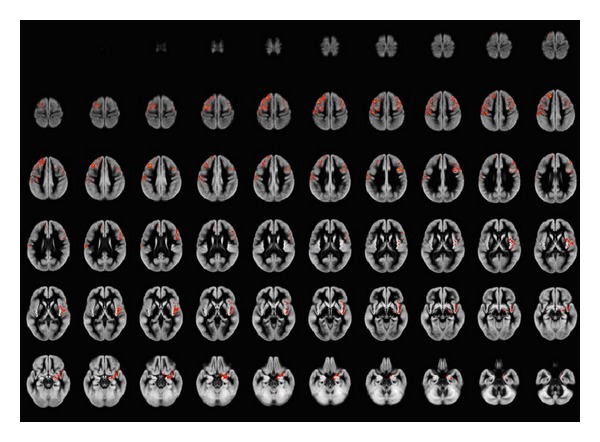
Areas of significant gray matter differences (including correction for age and total intracranial volume, less than 1 false-positive cluster expected, *P* < 0.001) across the three groups (normal volunteers and responder and nonresponder schizophrenia patients) at ANCOVA, superimposed onto the average of the normalized GM volumes. Right side of the brain is at the observer's right. GM differences among the three groups involve mainly the frontal lobes bilaterally. Superior and middle frontal gyri are involved on the left, extending into the pre- and postcentral gyri, while on the right mainly middle frontal gyrus and insula are involved. Additionally, right medial temporal lobe (mainly amygdala) is involved.

**Figure 2:**
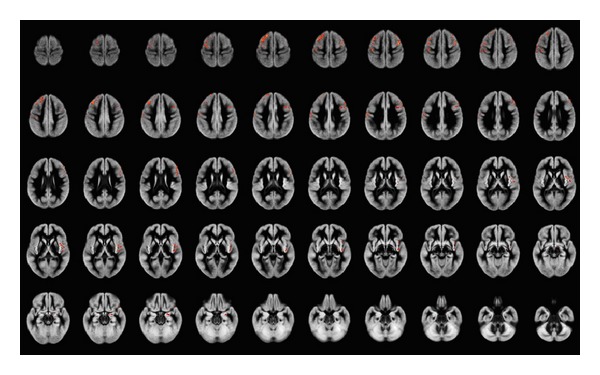
Areas of significant gray matter differences at ANCOVA (including correction for age and total intracranial volume, less than 1 false-positive cluster expected, *P* < 0.005) between normal volunteers and nonresponder schizophrenia patients, superimposed onto the average of the normalized GM volumes. Right side of the brain is at the observer's right. GM reduction in nonresponder schizophrenia patients involves mainly the frontal lobes bilaterally. Superior and middle frontal gyri are involved on the left, extending into the pre- and postcentral gyri, while on the right mainly middle frontal gyrus and insula are involved. Additionally, right medial temporal lobe (mainly amygdala) is involved.

**Figure 3:**
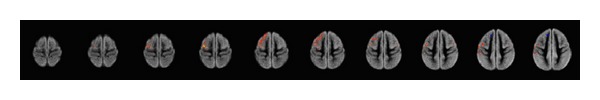
Areas of significant gray matter differences at ANCOVA (including correction for age and total intracranial volume, less than 1 false-positive cluster expected, *P* < 0.005) between normal volunteers and responder schizophrenia patients, superimposed onto the average of the normalized GM volumes. Right side of the brain is at the observer's right. Mainly left dorsal frontal cortex appears to be involved.

**Figure 4:**
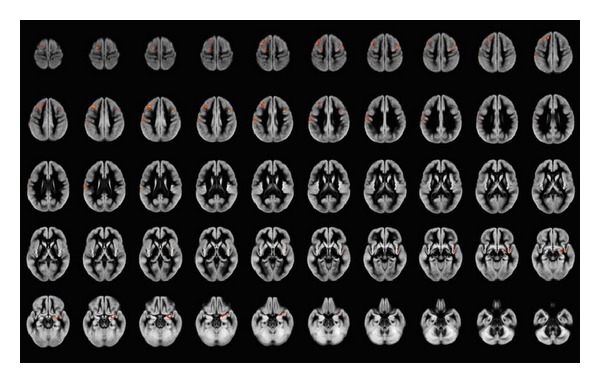
Areas of significant gray matter differences at ANCOVA (including correction for age and total intracranial volume, less than 1 false-positive cluster expected, *P* < 0.005) between responder and nonresponder schizophrenia patients, superimposed onto the average of the normalized GM volumes. Right side of the brain is at the observer's right. Clusters of significantly reduced GM volume in nonresponder schizophrenia patients compared to responder schizophrenia patients are detected mainly the frontal lobes bilaterally, more extended on the left where the pre- and postcentral gyri are involved, while on the right mainly middle frontal gyrus and insula are involved.

Clusters of significant GM differences among the three groups, as detected by morphometric analysis, are displayed in Figure 1. The results of the three post hoc comparisons (NV versus Resp-SC, NV versus NonResp-SC, and NonResp-SC versus Resp-SC) are shown in Figures 2, 3, and 4. Corresponding significant cluster size and locations are reported in Table 2.

Main differences between the three groups emerged bilaterally at the level of the superior and middle frontal gyri, with extension to the postcentral gyrus on the left and involvement of right insula and medial temporal cortex.

Post hoc analyses showed that these differences were due to a reduced GM volume of the frontal structures in NonResp-SC, compared to NV, while smaller clusters of significant GM loss were present in the R-CS compared to NV.

Finally, direct post hoc comparison of NonResp-SC and Resp-SC patients disclosed clusters of significantly reduced GM volume in NonResp-SC in superior and middle frontal gyri on the left, extending into postcentral cortex, and of middle frontal gyrus on the right, while smaller clusters (less than 0.5 cc) of reduced GM were detected in medial temporal lobe (mainly amygdala) and insula on the right.

No cluster of reduced GM volume was detected in NV versus both SC subgroups or in Resp-SC versus NonResp-SC.

## 4. Discussion

Our results demonstrate a significant reduction in GM volume in NonResp-SC mainly at the level of the superior and middle frontal gyri bilaterally and in right medial temporal cortex, as compared to Resp-SC.

Schizophrenic patients exhibit overall a decrease in brain volume in the temporal and frontal regions [42, 43]. A meta-analysis of gray matter anomalies in schizophrenia has shown that SC patients consistently show reduced gray matter density relative to control subjects in a network of regions, including bilateral insular cortices, anterior cingulate, left parahippocampal gyrus, left middle frontal gyrus, postcentral gyrus, and thalamus, coupled to increased gray matter density in striatal regions [44].

Previous neuroimaging studies using CT techniques have evaluated the correlation between frontal sulcal enlargement and poor clozapine response [14, 15, 18].

MRI transversal studies of treatment resistance in schizophrenia have shown a trend to a greater atrophy in NonResp-SC when assessing global brain tissue volumes [17]. In a study, the patients who required higher-doses of haloperidol had greater deficits in gray matter volume than those who were treated with the lowest dose [22].

Subsequent efforts to localize regions of atrophy, as assessed by manual segmentation of MRI studies, which correlate with the response to treatment, have highlighted a possible role of right prefrontal cortex in the response to clozapine [21] (although in the same study larger right prefrontal grey matter volume was associated with poorer response to haloperidol).

More recently a study, directly assessing structural parameters in patients resistant to conventional antipsychotics using Region of Interest (ROI) analysis of segmented MRI [20], has shown that NonResp-SC patients have significantly lower gray matter (GM) volumes in the frontal and occipital regions and significantly more white matter (WM) in the frontal, parietal, and occipital regions as compared to the controls, while these alterations are less prominent in Resp-SC patients, although in that case the direct comparison between NonResp-SC and Resp-SC patients failed to reach statistical significance.

In addition, in a recent study of morphometric correlates of response to atypical neuroleptics a direct comparison of baseline MR studies of Resp-SC versus NonResp-SC, has suggested that higher GM volumes in left rectus gyrus volumes are associated with a better acute response to treatment with risperidone or olanzapine [45], although in that case this difference did not survive correction for multiple comparisons.

When comparing these results to ours, possible effects of different treatment regimens should be considered. Besides the well-known effects of typical antipsychotics on basal-ganglia volume, in fact, apparent increases in GM have also been detected in occipital and parietal cortex during clozapine treatment in NonResp-SC [46] in the inferior frontal cortex/orbitofrontal gyrus and anterior cingulate cortex during quetiapine treatment [47] and in the cingulate gyrus for typical agents [48].

The effect of different treatment should be taken into account as in our case the two SC subgroups were under different therapeutic regimens, which may partly explain discrepancies with previous results of ROI-based analysis of baseline studies in patients treated with haloperidol only, which highlighted differences in occipital GM, beside frontal cortex [20], while in our case the presence of a substantial proportion of NonResp-SC undergoing clozapine treatment may have hindered parietal and occipital differences with the Resp-SC subgroup.

The heterogeneity of antipsychotics in our patient population precluded however an analysis of drug-specific effects.

Although previous data have reported an association between GM atrophy and increased number of hospitalization [49] with poorer overall outcome [50], possibly suggesting that ongoing neurodegenerative phenomena may partly cause (or be related to) the development of treatment resistance [51], the possibility that the increased cortical atrophy, mainly in frontal regions, is a consequence of the treatment with antipsychotics, rather than being related to the causes of treatment resistance, should be also considered.

Although frontal atrophy is a feature consistent across schizophrenia subsyndromes [52], influenced by the genetic background [53], and detectable also in patients' relatives [54], it has been indeed suggested that it can be exacerbated by treatment with antipsychotic, an hypothesis supported to some extent by meta-analyses of MRI segmentation studies [55, 56] although with differences between typical and atypical ones [49, 57, 58].

Furthermore, recent longitudinal studies in large cohorts of patients [58–60] have shown significant treatment effects on frontal GM atrophy, although without a significant treatment-by-time interaction [59], which would be expected if the cumulative effect of chronic therapy caused neurodegenerative phenomena.

Additionally, other factors should be considered when interpreting the meaning of structural differences in patient groups with a relatively large DD range, such as in our case. For example, differences of concurrent treatments between the two SC subgroups (antiepileptics and benzodiazepines being more frequently used in our NonResp-SC group of patients) may have also played a role in determining brain tissue volume differences, although the lack of a demonstrated persistent brain atrophy effect in chronic users of these classes of drugs [61, 62] mitigates against this hypothesis.

A limitation of the present study lies in the transversal approach in chronic patients, which precludes the possibility of extending the conclusions to first-episode patients, thus leaving open the question whether the more pronounced frontal GM loss is a cause or a consequence of the treatment resistance.

In this respect it is of note that an MRI study in a large group of first-episode schizophrenia or schizoaffective disorder patients has not detected a significant effect of MRI variables onto prediction of treatment response [63], although in that case semiautomated ROI-based analysis of predefined brain structures, not covering the frontal lobes, was used.

Finally, for the purposes of the present study we dichotomized our patient population. However, the concept of treatment resistance may be recognized as a continuum rather than a dichotomy of response versus nonresponse [64], so that a correlation analysis in a larger cohort of patients could provide additional information.

## 5. Conclusions

We have shown significant structural cerebral differences in a direct comparison between two groups of NonResp-SC and Resp-SC patients, suggesting an association between bilateral frontal and right medial temporal cortex alterations, and treatment resistance in schizophrenia. The results of the present study need to be confirmed in longitudinal studies in drug-naïve, first-episode patients, to assess the role of these alterations and their possible predictive value.

## Figures and Tables

**Table 1:** Demographic and clinical data.

		Age	DD	AAO	Antipsychotics∗	E-BPRS	BPRS-PS	BPRS-NS
NV (*n* = 16)	Mean	35.2	n/a	n/a	n/a	n/a	n/a	n/a
	SD	11.3	n/a	n/a	n/a	n/a	n/a	n/a
	Median	35.8	n/a	n/a	n/a	n/a	n/a	n/a
	Min.	21.1	n/a	n/a	n/a	n/a	n/a	n/a
	Max.	57.0	n/a	n/a	n/a	n/a	n/a	n/a

Resp-SC (*n* = 15)	Mean	33.5	11.1	22.3	310.6^§^	26.9^§^	5.7^§^	3.3^§^
	SD	11.2	9.6	9.4	300	3.8	1.2	0.6
	Median	28.8	8	20.2	239.9	25	5	3
	Min.	20.5	1	12.5	60	24	5	3
	Max.	54.6	30	48.5	1008	35	9	5

NonResp-SC (*n* = 20)	Mean	33.1	13.0	20.1	591.4	73.3	23.05	8.55
	SD	7.6	6.8	3.2	525	9.2	4.3	3.3
	Median	33.8	13	20.1	331.3	76.5	24.5	8.5
	Min.	19.2	1	14.2	120	49	11	4
	Max.	51.7	30	26.6	1350	86	28	17

DD: Disease Duration (years).

AAO: Age at onset (years).

E-BPRS: Expanded Brief Psychiatric Rating Scale, version 4.0.

BPRS-PS: BPRS Positive Symptoms.

BPRS-NS: BPRS Negative Symptoms.

n/a: not applicable.

∗Current daily dose of antipsychotics converted in mg equivalents of chlorpromazine [23].

^§^Significatly different between Resp-SC and NonResp-SC (*P* < 0.005 at Mann-Whitney test).

For E-BPRS BPRS-PS, and BPRS-NS (higher scores indicate more severe psychiatric symptoms).

**Table 2:** Regional gray matter differences across the three groups.

Region	Side	Omnibus	POST-HOC		
			Resp-SC < NV	NonResp-SC < NV	NonResp-SC < Resp-SC
Postcentral gyrus	Left	2.2		2	0.8
Precentral gyrus	Left	1.5	0.6	1.4	
Superior frontal gyrus, dorsolateral	Left	2.4		1.6	0.9
Middle frontal gyrus	Left	3.1	0.6	2.9	1.5
Precentral gyrus	Right	1		0.8	
Rolandic operculum	Right	0.5		0.5	
Middle Frontal gyrus	Right	2.2		2.2	0.6
Inferior frontal gyrus, opercular part	Right	1.1		1	
Inferior frontal gyrus, triangular part	Right	0.7		0.6	
Insula	Right	2.2		2.2	
Amygdala	Right	0.7		0.7	

Volumes (in cc) of the clusters of significant gray matter differences (less than 1 false-positive cluster expected) across the three groups of patients at ANCOVA (omnibus test) and at the three post-hoc tests.

Localization is according to Tzourio-Mazoyer, et al. [24].

Structures involved for less than 0.5 cc are not listed.

NV: Normal Volunteers.

NonResp-SC: Non-Responder schizophrenia patients.

Resp-SC: Responder schizophrenia patients.

## List of Abbreviations

AAO: Age at Onset
ANCOVA: Analysis of covariance
BPRS-NS: BPRS negative symptoms
BPRS-PS: BPRS positive symptoms
CSF: Cerebrospinal fluid
DD: Disease duration
E-BPRS: Expanded brief psychiatric rating scale
GM: Gray matter
ICV: Intracranial volume
MNI: Montreal neurological institute
NonResp-SC: Nonresponder schizophrenia
NV: Normal volunteers
ROI: Region of interest
Resp-SC: Responder schizophrenia
SC: Schizophrenia
WM: White matter.
